# Impact of Cesium Concentration on Optoelectronic Properties of Metal Halide Perovskites

**DOI:** 10.3390/ma15051936

**Published:** 2022-03-04

**Authors:** Steponas Ašmontas, Aurimas Čerškus, Jonas Gradauskas, Asta Grigucevičienė, Remigijus Juškėnas, Konstantinas Leinartas, Andžej Lučun, Kazimieras Petrauskas, Algirdas Selskis, Algirdas Sužiedėlis, Edmundas Širmulis

**Affiliations:** Center for Physical Sciences and Technology, Savanorių Ave. 231, LT-02300 Vilnius, Lithuania; aurimas.cerskus@ftmc.lt (A.Č.); jonas.gradauskas@ftmc.lt (J.G.); asta.griguceviciene@ftmc.lt (A.G.); remigijus.juskenas@ftmc.lt (R.J.); konstantinas.leinartas@ftmc.lt (K.L.); andzej.lucun@ftmc.lt (A.L.); kazimieras.petrauskas@ftmc.lt (K.P.); algirdas.selskis@ftmc.lt (A.S.); algirdas.suziedelis@ftmc.lt (A.S.); edmundas.sirmulis@ftmc.lt (E.Š.)

**Keywords:** perovskite, cesium, solar cell, thin film, power conversion efficiency

## Abstract

Performance of a perovskite solar cell is largely influenced by the optoelectronic properties of metal halide perovskite films. Here we study the influence of cesium concentration on morphology, crystal structure, photoluminescence and optical properties of the triple cation perovskite film. Incorporation of small amount (x = 0.1) of cesium cations into Cs_x_(MA_0.17_FA_0.83_)_1−x_ Pb(I_0.83_Br_0.17_)_3_ leads to enhanced power conversion efficiency (PCE) of the solar cell resulting mainly from significant rise of the short-current density and the fill factor value. Further increase of Cs concentration (x > 0.1) decreases the film’s phase purity, carrier lifetime and correspondingly reduces PCE of the solar cell. Higher concentration of Cs (x ≥ 0.2) causes phase segregation of the perovskite alongside with formation of Cs-rich regions impeding light absorption.

## 1. Introduction

Metal halide perovskites attract considerable attention due to their remarkable properties revealing potential applications in optoelectronic devices such as solar cells [1,2,3,4,5,6,7,8,9,10,11,12,13], photodetectors [14,15,16,17,18,19,20,21,22,23], light emitting diodes [24,25,26,27,28,29,30,31,32,33,34]. They have also been used for photocatalysts [35] and bioimaging [36]. In solar cells, metal halide perovskites are attractive for their unique features such as high value of the absorption coefficient across the entire visible spectrum range that allows to use a thin perovskite film [37,38], high defect tolerance [39], high carrier mobility [40,41], and long carrier diffusion length [42,43]. Possibility of fine band gap tuning allows using perovskite layer as a top sub-cell in a tandem solar cell [44,45,46]. The methylammonium (MA) based metal halide perovskite has been the pioneer in this class of materials. However, MA cation-based metal halide perovskites suffer from inherently high instability when exposed to heat or moisture [8,12]. It was shown that degradation of MAPbI_3_ perovskites caused by illumination or elevated temperatures is typically triggered by the volatilization of the MA cation [12]. In search of stable perovskite crystal, cesium [47] or formamidinium (FA) was used in combination with the MA cation [44,47,48]. At present, the best quality demonstrating solar cells use a mixture of (FA) and (MA) as the monovalent cations [48,49,50,51,52,53,54]. Addition of cesium makes the triple cation perovskite composition thermally more stable as far as it has less phase impurities and is less sensitive to processing conditions [48,55,56]. It is shown that incorporation of small amount of Cs reduces the trap density and charge recombination rate in the perovskite layers [57]. Thus, performance of the double cation perovskite solar cells can be improved by means of adding a small amount of Cs cations [49,56,57,58,59]. Addition of Cs also enhanced the quality of formamidinium lead iodide perovskite solar cells; this gain was explained by the improved crystallinity which is linked to stabilization of the photoactive perovskite layer by suppressing the phase transition into the yellow nonperovskite structure at room temperature [59,60,61,62]. Rehman et al. demonstrated that charge carrier mobility and lifetime were increased substantially due to addition of Cs into the mixed-cation lead mixed-halide perovskite Cs_x_FA_1−x_ Pb(I_0.6_Br_0.4_)_3_ film in relative content of Cs up to x = 0.2, and then was followed by a plunge of these parameters to much lower values in the content range of 0.3 < x < 0.8 [61]. Since the mixture of (FA) and (MA) perovskite layers is widely used as a top sub-cell in the tandem solar cells [45,46,47,48,49,63,64,65,66,67,68,69,70,71,72,73,74], it is essential to understand the impact of cesium cation additive on perovskite’s optoelectronic properties aiming at improved performance of tandem solar cells.

In this work, we present the results of experimental study of optical, photoluminescence and charge transport properties of perovskite Cs_x_(MA_0.17_FA_0.83_)_1−x_ Pb(I_0.83_Br_0.17_)_3_ layers containing different cesium concentration (0 ≤ x ≤ 0.6). The transient photovoltage and photoelectric characteristics of the perovskite solar cells are reported as well.

## 2. Fabrication of Perovskite Films and Their Characterization

**Figure 1:**
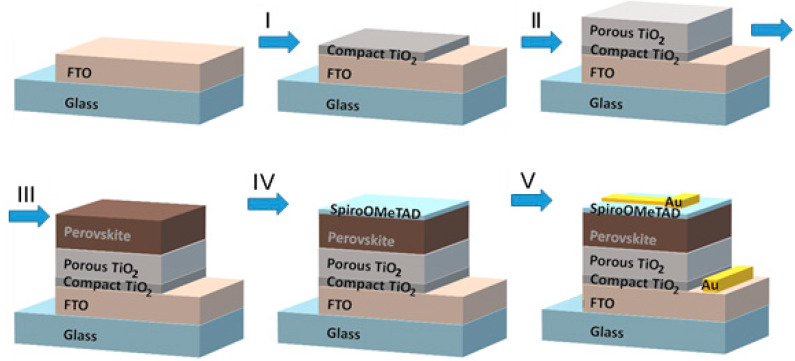
Schematic step-like representation of the perovskite solar cell formation.

In the present study, perovskite films were fabricated according to the methods described earlier [49,75,76]. All the reagents, their concentrations and purity grades, the sequence of procedures and equipment used for the fabrication of the perovskite solar cells were depicted in detail in our previous paper [58]. The triple cation perovskite films were deposited on the 25 × 25 mm^2^ glass substrates coated with transparent fluorine-doped tin oxide (FTO) layer (TEC 10, Ossila B.V., Sheffield, UK). Approximately 7 × 25 mm^2^ of the FTO coating was removed from one edge of the glass substrate using zinc powder (Sigma Aldrich, MO, USA) and hydrochloric acid (Merck KGaA, Darmstadt, Germany), followed by sonication in 2% Helmanex (Hellma GmbH&Co, Müllheim, Germany) for 20 min. Then the substrates were rinsed with deionized water and sonicated in isopropanol (Merck KGaA, Darmstadt, Germany) for 20 min before being exposed to plasma treatment for 10 min. The main steps of the perovskite solar cell formation are shown in Figure 1.

During step I, a ~30 nm-thick compact TiO_2_ (Greatcell Solar Italia S.R.L., Roma, Italy) layer was deposited on the FTO coating by means of spray pyrolysis of precursor solution consisting of titanium diisopropoxide (bis) acetylacetonate Ti(acac)_2_OiPr_2_ (Merck KGaA, Darmstadt, Germany) and anhydrous ethanol (Sigma Aldrich, MO, USA) (1:9 volume ratio) at 450 °C followed by annealing for 15 min. After the deposition, the structures were allowed to cool down to room temperature. During step II, mesoporous TiO_2_ layer was formed by spin coating of 180 µL of suspension containing titanium oxide nanoparticles (30 nm) diluted in ethanol (1:6 weight ratio) at 4000 rpm for 20 s with an acceleration of 2000 rpm·s^−2^. Then the substrates were annealed at 450 °C for 30 min. After cooling down, they were transferred to a nitrogen-filled glove-box (M. Braun Inertgas-Systeme GMBH, Garching bei München, Germany). During step III, the perovskite layer was deposited from a freshly prepared solution of 1.2 M lead iodide (Sigma Aldrich, MO, USA), 0.2 M methylammonium bromide (Greatcell Solar Italia S.R.L., Roma, Italy), 0.2 M lead bromide (Sigma Aldrich, MO, USA) and 1 M formamidinium iodide (Greatcell Solar Italia S.R.L., Roma, Italy). The mixture of anhydrous N,N-dimethylformamide (Sigma Aldrich, MO, USA), and dimethylsulfoxide (DMF/DMSO) (Sigma Aldrich, MO, USA) with volume ratio of 4:1 was used as a solvent. The content of cesium ions in the perovskite layer was varied by means of choosing CsI (Strem Chemicals INC MS, IL, USA) (1.5 M of CsI in DMSO) concentration from 0 to 60%. The prepared precursor solution was spin coated by a two-step program: the first was at 1000 rpm for 10 s, and the second was at 6000 rpm for 30 s. Approximately 10 s prior to the end of the spinning, 150 µL of chlorobenzene (Sigma Aldrich, MO, USA) was dropped onto the top surface and annealed at 100 °C for 60 min. The fourth step of the cell fabrication was up for formation of the hole transporting layer (HTL). The HTL was formed by spinning of 150 μL of 70 mM solution of 2-*N*,2-*N*,2-*N*′,2-*N*′,7-*N*,7-*N*,7-*N*′,7-*N*′-octakis(4-methoxyphenyl)-9,9′-91 spiro-bi[fluorene]-2,2′,7,7′-tetramine (Spiro-OMeTAD) in chlorobenzene (85.78 mg/mL), supplemented with 17 μL of Li-bis((trifluoromethyl)sulfonyl)imide (Li-TFSI) salt in anhydrous acetonitrile (520 mg/mL) and 28.8 μL of 4-tert-butylpyridine (TBP) solutions just before the application (all from Sigma Aldrich, MO, USA). The molar ratio of dopants to the Spiro-OMeTAD was 0.5 and 3.3 for Li-TFSI and 4TBP, respectively. The spin-coating regime of step IV was 4000 rpm for 25 s with acceleration of 2000 rpm·s^−2^. During the final step V, ~70 nm-thick Au contacts (Kurt J. Lesker Company, Clairton, Germany) were deposited on top of Spiro-OMeTAD and FTO by means of thermal evaporation in the vacuum chamber of “VAKSIS PVD Vapor-5S_Th” (Vaksis R&D and Engineering, Ankara, Turkey).

Morphology and thickness of the perovskite films were examined by means of scanning electron microscope (SEM) (Helios NanoLab 650, FEI, Hillsboro, OR, USA). Chemical composition of the films was studied with the SEM-supplemented energy dispersive X-ray spectrometer (EDX) (INCA Energy, Oxford Instruments, Abingdon, UK).

Crystallographic structure of the fabricated perovskite films was investigated using X-ray diffractometer (XRD) equipped with a 9 kW power rotating Cu anode X-ray source and theta/theta goniometer (SmartLab, Rigaku, Tokyo, Japan). The patterns were measured with Bragg-Brentano geometry in 2Θ range of 10°–65°. The main features of the full-scale perovskite layers’ XRD patterns were analyzed in our previous work [55]. In this study, the detailed analysis of the fragments of XRD patterns in the 2Θ range of 10–15° was carried out.

Photoluminescence (PL) and transient PL spectra were measured at room temperature. An Ar-ion laser or diode pumped solid state (DPSS) microchip laser (Standa Ltd., Vilnius, Lithuania) were used for excitation, and a 1-m monochromator and TE-cooled PMT utilizing photon counting technique were used for data recording. More details of the technique were described in our previous paper [58]. The Ar-ion laser power density was varied over three orders of magnitude (from 63 down to 0.06 mW/cm^2^) and the microchip laser fluence was varied over two orders of magnitude, from 0.076 to 7.6 μJ/cm^2^ per pulse, with a linear variable metallic neutral density filter.

Transient photovoltage of the solar cells was investigated using 7 ns-long laser pulses of 532 nm wavelength generated at 50 Hz repetition rate by the diode pumped frequency-doubled Nd:YAG-LBO laser NL202 (Ekspla Ltd., Vilnius, Lithuania). Average power of the laser radiation was measured by optical power meter PM400 (Thorlabs Inc., Newton, NJ, USA). The transient photovoltage and the laser pulse were recorded by digital storage oscilloscope Agilent Technologies DSO6102A (Agilent Technologies Inc., Santa Clara, CA, USA), and the laser pulse shape was registered by the high-speed optical signal reference detector 11HSP-FS1 (Standa Ltd., Vilnius, Lithuania).

Absorbance spectra of the perovskite films were estimated from the optical transmission data obtained with spectrometer AvaSpec-ULS2048XL-EVO (Avantes, Apeldoorn, The Netherlands).

Keithley 2602A (Keithley Instruments Inc., Cleveland, OH, USA) equipment was used for direct measurement of current-voltage characteristics of the perovskite solar cells. The 100 mW/cm^2^ irradiance was achieved by an AM 1.5 spectral lamp (Newport model 67005, Newport Corp., Irvine, CA, USA) placed at an appropriate distance.

## 3. Results and Discussion

**Figure 2:**
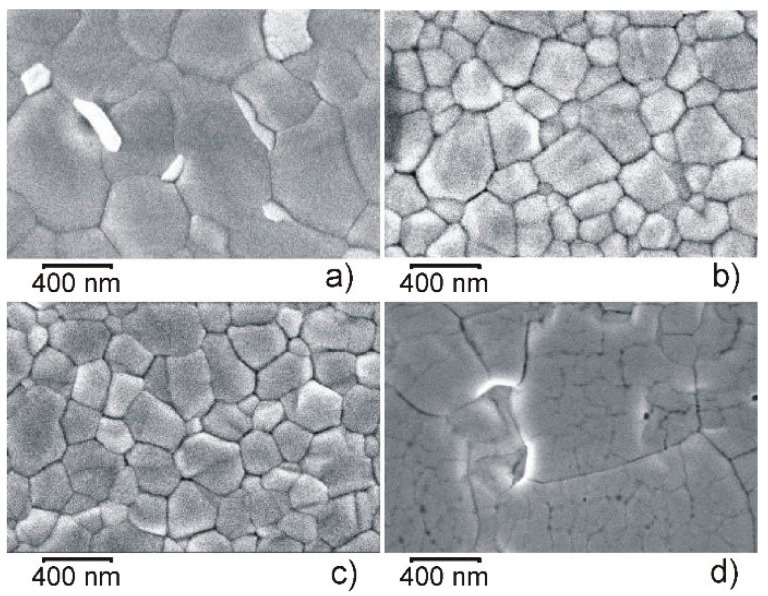
Surface SEM images of the formed perovskite films. Cs concentration (in %) is: (**a**) 0, (**b**) 10, (**c**) 20, (**d**) 40. Magnification is 100,000×.

The grain size and morphology of the perovskite films grown on mesoporous TiO_2_ scaffolds were strongly dependent on cesium concentration in the precursor solution. The top-view SEM images of the triple cation perovskite films prepared from solution containing different concentration of Cs (0, 10, 20, and 40%) are shown in Figure 2.

**Figure 3:**
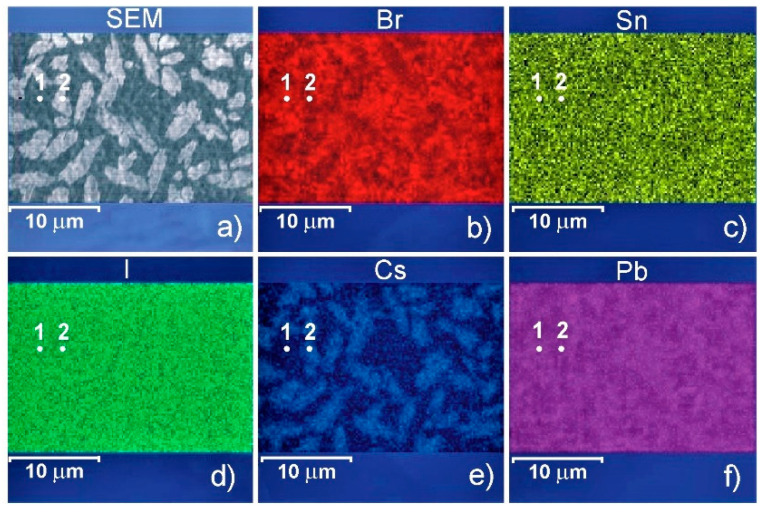
Surface SEM image (**a**) and mapping images of EDX analysis of 30% CsI containing perovskite layer corresponding to bromine (**b**), tin (**c**), iodine (**d**), cesium (**e**), and lead (**f**) mapping.

It is seen that morphology of the perovskite layer without cesium (0 Cs) can be characterized by formation of big grains of ~400–900 nm in size. It is worth noting that in this case (perovskite without Cs) significantly smaller grains which are attributed to an individual PbI_2_ phase tend to be formed at the perovskite grains’ boundaries [55]. Addition of CsI to the precursors solution and resulting incorporation of Cs^+^ ions into the layer structure lead to formation of considerably smaller perovskite grains because Cs-induced perovskite seeds become nucleation sites for further crystal growth and thus lead to more uniform grains [49,55]. When the layers contain 10 and 20% of Cs, the grains of ~100–200 nm in size prevail, and their boundaries are clearly distinct. Futher increase of CsI content in the solution leads to the change in the morphology of the formed perovskite layers: the grains show a tendency to form larger agregates, they seem to be more integral, i.e., their boundaries are not so clearly distinct (Figure 2, 40 Cs). At higher concentration of Cs (more than 20%), not all Cs ions get into the perovskite lattice, and their excess can form a mixed compound with Pb (as a separate phase on the surface of the layer) [77] and/or is flushed out by chlorobenzene in step III of the fabrication. Distribution of Cs on the surface of perovskite layer can be clearly seen in Figure 3 where the EDX mapping is presented.

As Figure 3 shows, the EDX images confirm that Br, Sn, I, and Pb elements are homogeneously dispersed while Cs is segregated into 2–5 m-size islands. The details of the elemental composition of the sample surface at points 1 and 2 are presented in Table 1.

**Figure 4:**
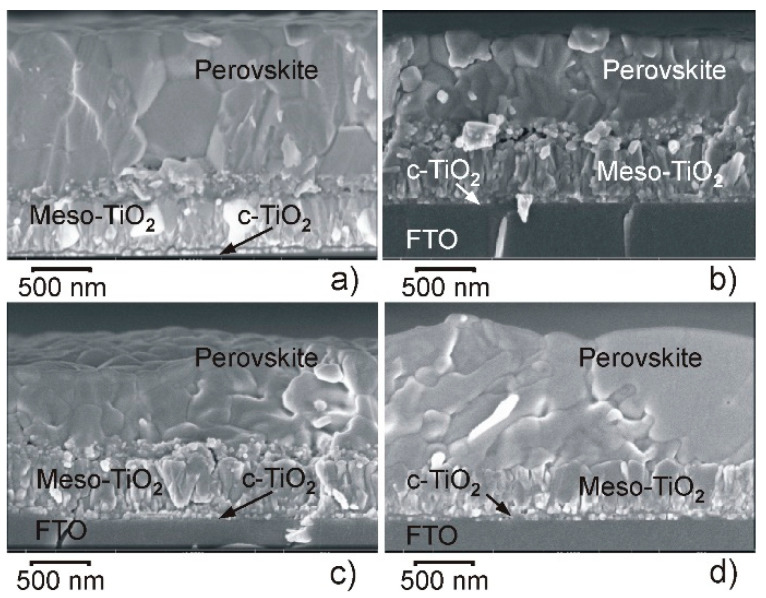
Cross-sectional SEM images of the fractures of the perovskite cells. Cs concentration (in %) is: (**a**) 0, (**b**) 10, (**c**) 20, (**d**) 40. Magnification is 65,000×.

Morphological changes of the same perovskite samples are significantly less pronounced in the cross-sectional SEM images (Figure 4). At higher Cs concentration (20% and 40%), the perovskite layers look amorphous since crystals or crystalline aggregates (conglomerates) are hardly visible (Figure 4c,d). The thickness of the perovskite films grown on meso-TiO_2_ is in the range of 850 nm to 1100 nm.

**Figure 5:**
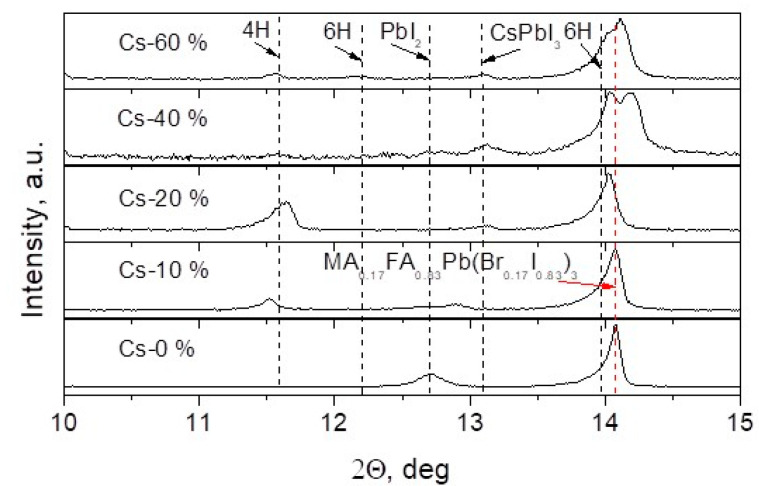
Fragments of XRD patterns of perovskite films formed with different concentration of Cs in the precursor solution.

Influence of Cs on structure and phase composition of the formed perovskite layers was studied by the XRD technique with Cs concentration ranging from 0 to 60%. Figure 5 shows fragments of the measured XRD patterns.

No Cs containing film demonstrated XRD peaks that could be attributed to MA_0.17_FA_0.83_Pb(Br_0.17_I_0.83_)_3_ phase with cubic crystal lattice (a = 6.284 Å) and to PbI_2_ which are both characteristic of perovskite films without Cs^+^ ions. Addition of 10% of CsI into the precursor solution resulted in vanished PbI_2_ phase. However, additional peaks emerged at 2Θ angles of 11.53° and 12.87°. The last peak could be attributed to possible solid solution of Pb_x_Cs_x−1_I_2_ since the replacement of Pb ions by those of smaller ionic radius Cs should cause a shift of peak towards bigger 2Θ angles. In case of 20% of Cs containing film, rather strong peak at 2Θ angle of 11.6° emerged on the XRD pattern, and the peak of the perovskite phase was shifted to lower diffraction angles. This could be caused by the presence of hexagonal (P6_3_/mmc) polytypes of the perovskite [78]. The low intensity peak at 2Θ angle of 13.06° indicated presence of small amount of CsPbI_3_ in the film. Bigger quantity of Cs^+^ ions caused formation of two perovskite phases with cubic structure since each perovskite peak on the XRD pattern split into two peaks. The structure with larger crystal lattice (a = 6.304 Å) showed lower volume percentage while that with smaller lattice (a = 6.241 Å) was bigger in quantity according to higher intensity of the corresponding peaks. Decrease of the perovskite lattice parameter most probably results from the replacement of MA^+^ ions by those of smaller radius Cs^+^. Increase of the lattice parameter could be caused by the rise of I/Br or/and FA/MA ratios in the perovskite crystal lattice. On the other hand, the peak at 2Θ angle of 14.0° could be related to the 6H polytype of the perovskite [78]. It means that high Cs concentration leads to structural inhomogeneities, crystal cleavages, and phase segregation, all these being detrimental to device performance and potentially serving as recombination centers [79].

**Figure 6:**
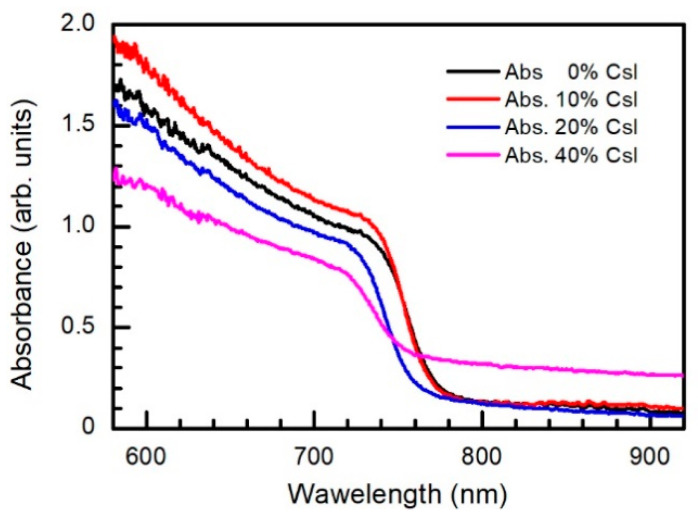
Absorbance spectra of perovskite films with different Cs concentration.

The measured absorbance spectra of the perovskite Cs_x_(MA_0.17_FA_0.83_)_1−x_ Pb(I_0.83_Br_0.17_)_3_ films with different cesium concentration are shown in Figure 6.

It is seen that introduction of Cs into the perovskite causes gradual blue-shift of the absorption spectrum. When small amount of Cs (x ≤ 0.1) is introduced, absorbance of the perovskite layer in the visible spectrum range increases, and then begins to decrease at higher cesium concentration (x > 0.1). The observed weakening of the absorption with higher cesium concentration seems to be stipulated by segregation of CsPbI_3_ phases or 6H polytype of perovskite as mentioned earlier. Decrease of the absorbance of the perovskite layer with higher Cs concentration was previously observed in other works [49,79]. It should be noted that absorption of the infrared spectrum range in 40% of Cs-containing perovskite layer is high enough due to strong scattering of light by structural inhomogeneities, crystal cleavages, and phase segregation [79].

**Figure 7:**
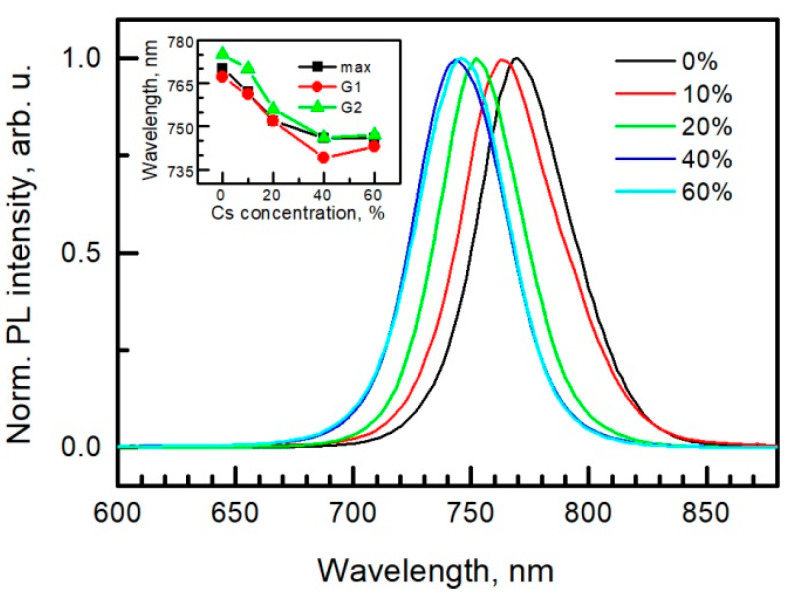
PL spectra of perovskite layers with different cesium concentration. The inset shows position of the PL peak maximum and two Gaussian peaks versus Cs concentration.

Photoluminescence spectra of the perovskite layers are shown in Figure 7. Increase in Cs concentration shifts PL maximum to the blue side. However, the red shift is detected in the sample with the highest, 60%, concentration. Similar tendencies were observed in references [49,80]. This red-shift could be explained by the presence of the second cubic or hexagonal 6H polytype of perovskite as mentioned above.

Each peak (*λ_max_* is the wavelength of the peak maximum) was convoluted by the two-amplitude version of the Gaussian function $y\left(\lambda \right)=A_{i}e^{-\frac{{\left(\lambda -\lambda_{ci}\right)}^{2}}{2\omega_{i}^{2}}}$, where *λ_ci_* is the peak center, *ω_i_* is the width (full width at half maximum $y\left(\lambda \right)=A_{i}e^{-\frac{{\left(\lambda -\lambda_{ci}\right)}^{2}}{2\omega_{i}^{2}}}$, and *A_i_* is the height of the Gaussian peaks. Results of the best convolution are shown in the inset of Figure 7 and Table 2.

**Figure 8:**
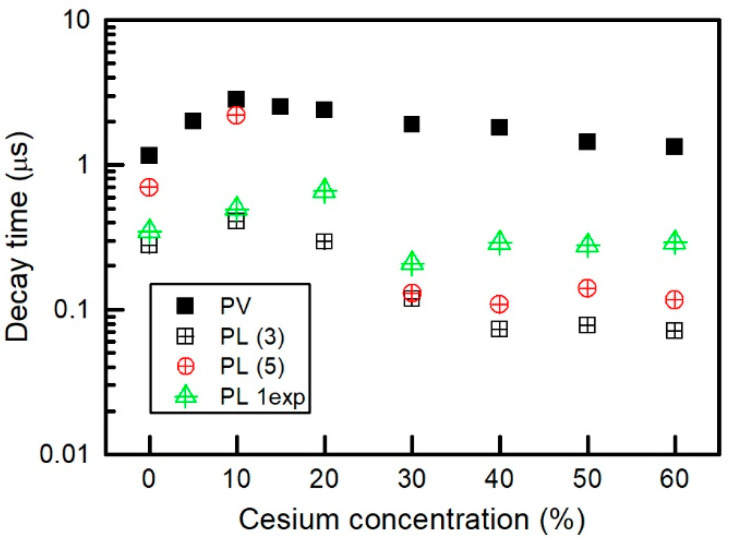
Dependence of photovoltage (PV) and photoluminescence (PL) decay time constant on cesium concentration in the perovskite layer.

Gaussian peaks G1 and G2 could be related to band–band and excitonic transitions in the samples with lower Cs concentration. Meanwhile, in the samples with 20% and 60% of Cs, peak G2 could be related to the transitions of two different phases due to the decreased difference between the peak centers in accordance with the XRD results. A Tauc plot was used to determine the optical band gap. These values and corresponding wavelengths are presented in Table 2. One can observe good correlation between the band gap wavelength and Gaussian peak G1, except the cases of 40 and 60 percent of Cs. The latter discrepancy is also related to the presence of the second cubic or hexagonal 6H polytype of the perovskite. Another possible explanation of the PL spectrum asymmetry could be related to spatial potential fluctuations and to transitions from band to the tail of the localized states. This asymmetric spectrum can be fitted by a double sigmoid function [81]. We used it in the form of asymmetric

(1) $$y\left(\lambda \right)=A\frac{1}{1+e^{-\frac{\lambda -\lambda_{c}+\omega /2}{\omega_{1}}}}\left(1-\frac{1}{1+e^{-\frac{\lambda -\lambda_{c}-\omega /2}{\omega_{2}}}}\right),$$

where *A*, *λ_c_*, *ω* is the maximum amplitude, the center and the width of the curve, respectively; and *ω*_1_*, ω*_2_ are the shape parameters of short- and long-wavelength side. Fitting results of Equation (1) are presented in Table 2. Changes of the width *ω* and long-wavelength side shape parameter *ω_2_* correlate with the changes of the average decay times (see Table 2 and Figure 8).

**Figure 9:**
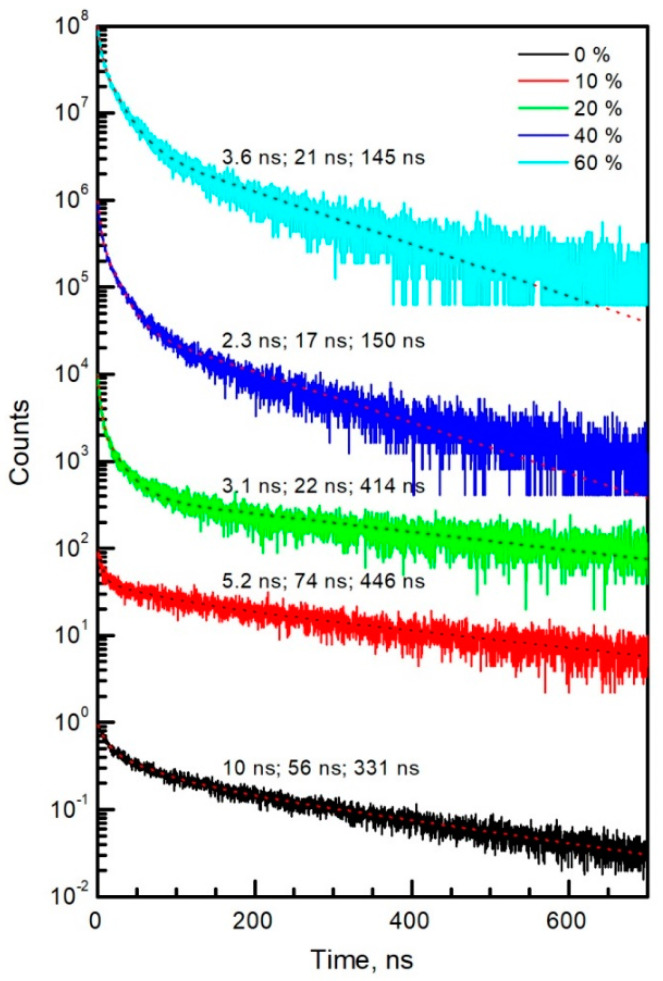
PL decay transients of the perovskite layers with different cesium concentration. Dotted lines are fittings using Equation (2) with *i* = 3.

The transient PL curves measured in the peak center were fitted using multi-exponential model

(2) $$I_{\mathrm{PL}}\left(t\right)=\sum_{i}A_{i}e^{-\frac{t}{\tau_{i}}},$$

where *τ_i_* and *A_i_* are the decay time constant and the amplitude of the *i*th component, respectively. However, only at least three exponents provided good fitting, and these results are shown in Figure 9 with corresponding time constant values.

Two of the decay time constants we assign to the transitions related with the Gaussian peaks. The third decay time could be related with various nonradiative processes, or the longest decay time could be attributed to carrier trapping and detrapping processes [82]. Decrease of the decay time constants with higher Cs concentration we relate with increased number of defects and surface recombination. As a rule, low purity samples have lower values of the decay constants [83]. The average decay time constant was calculated as

(3) $$\bar{\tau }=\frac{\sum_{i}A_{i}\tau_{i}^{2}}{\sum_{i}A_{i}\tau_{i}}$$

and was compared with the decay time constants obtained from other fittings and from the transient photovoltage measurement (see Figure 8).

Notwithstanding the application of the three decay times, one cannot explain the case of transition from band to localized states’ tail even by taking into account the trapping-detrapping processes. Thus, we fitted the transients with more general decay function encompassing both the stretched exponential and the compressed hyperbola function [84] as

(4) $$I_{\mathrm{PL}}\left(t\right)=Ae^{\frac{1-{\left(1+\alpha \frac{t}{\tau_{0}}\right)}^{\beta }}{\alpha \beta }},$$

where *α* and *β* are the dimensionless parameters. Then the average decay time constant was calculated numerically using the following equation:

(5) $$\bar{\tau }=\frac{\int_{0}^{\infty }tI_{\mathrm{PL}}\left(t\right)dt}{\int_{0}^{\infty }I_{\mathrm{PL}}\left(t\right)dt}.$$

The fitting results are also presented in Table 2 and Figure 8. The averaged decay time constants obtained by means of Equations (3) and (5) show the same tendency; see, respectively, PL (3) and PL (5) in Figure 8. The only exception is the sample with 20% of Cs whose averaged decay time constant given by Equation (5) goes to infinity. For comparison, we extracted one-exponent decay time constant ranging from 400 ns up to 700 ns (see PL 1 exp in Figure 8).

**Figure 10:**
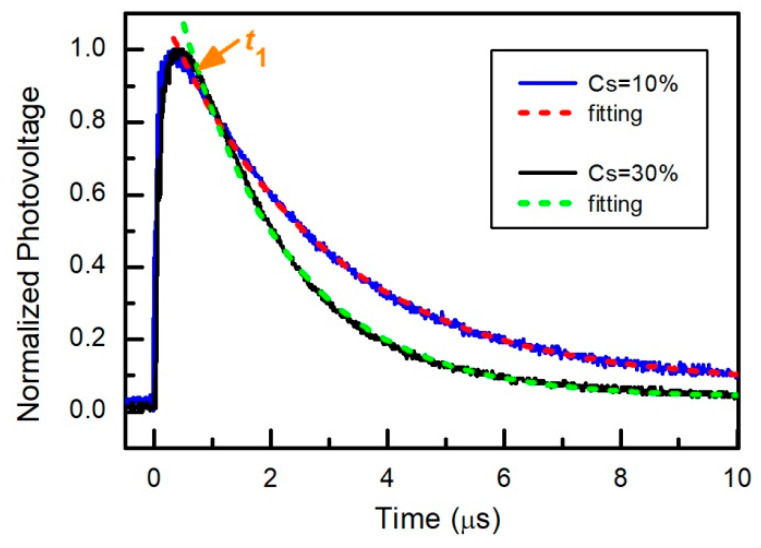
Photovoltage decay transients of the solar cells with different concentrations of Cs in perovskite layer. Excitation laser power density is 1.4 mW/cm^2^.

Figure 10 shows transients of photovoltage measured across the perovskite solar cell with different cesium concentration.

It is seen that, in a certain interval of time (*t* > *t*_1_), the decay of the photovoltage can be approximated as a simple exponential function $U_{\mathrm{ph}}=U_{\mathrm{ph}0}e^{-t/\tau }$, where *U*_ph0_ is the value of *U*_ph_ at time *t*_1,_ and *τ* is the characteristic decay time constant. The dependence of the transient photovoltage time constant on cesium concentration is depicted in Figure 8. PV and PL decay times increase only after addition of small amount of Cs, and later start decreasing. This dependence can be explained by the change in the layer quality that leads to reduction of the trap density and charge recombination rates in the perovskite layers [57]. Adding of Cs firstly minimizes the quantity of PbI_2_ phase, but later there appears CsPbI_3_ and other polytypes of the perovskite (see Figure 5). It is seen that all the photovoltage time constants have values higher than those obtained from the photoluminescence measurements. This difference could be explained by several reasons [85]. First, the real bulk carrier lifetime is masked by the combination of the surface recombination and self-absorption of reemitted photons in the volume of the perovskite layer. Therefore, the PL decay time can be shorter by an order of magnitude than the real bulk lifetime. Second, the photovoltage decay reflects the bulk recombination processes and produces a proper value of the latter. Third, in the case of a thin film, the transient photovoltage decay is strongly extended by the low ambipolar mobility resulting from the space charge-limited currents.

**Figure 11:**
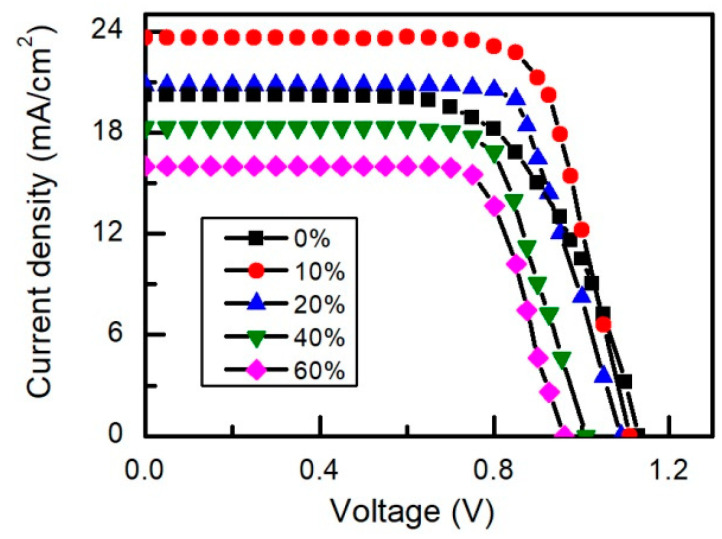
Current voltage characteristics of perovskite solar cells with different cesium concentration. Light intensity is 100 mW/cm^2^ of AM 1.5 spectral lamp.

Figure 11 shows current-forward bias voltage characteristics of the fabricated perovskite solar cells with different cesium concentration. As expected, the cell containing 10% of cesium in the perovskite layer has the highest value of the short-current density *J_sc_*. This is the result of the highest absorbance of the 10%-perovskite layer. This sample also demonstrates the highest values of the photovoltage and the PL decay time constants. It is worth noting that the current-voltage characteristics of solar cell containing 10% of Cs shows negligible hysteresis, just like it was observed earlier in References [49,58,75]. Significant hysteresis of the characteristic was observed only in the case of high cesium concentration (≥20%). This behavior might be related to the phase segregation within the cubic perovskite phase and to reduced crystallinity [79,80].

**Figure 12:**
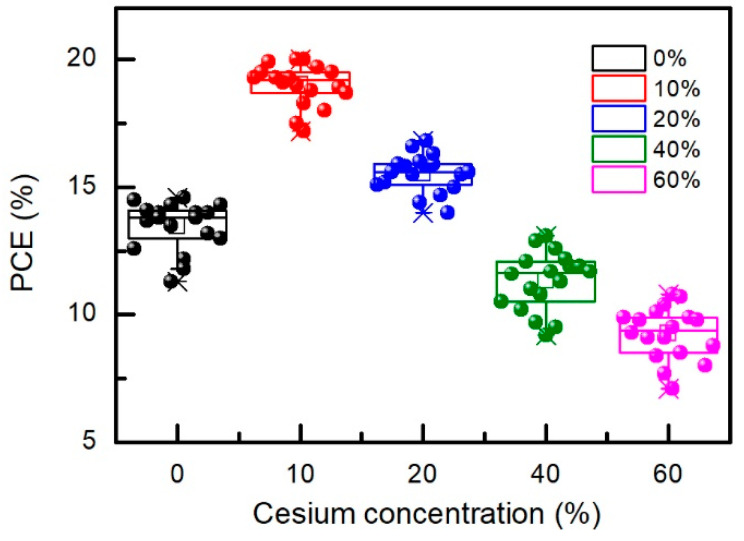
Statistical distribution of power conversion efficiency of perovskite solar cells versus Cs concentration. Error bars indicate plus or minus one standard deviation from the mean.

The values of photovoltaic quantities of the best perovskite solar cells are presented in Table 3, and statistics of the power conversion efficiency of 90 perovskite solar cells with different cesium concentration is depicted in Figure 12.

The run of PCE dependence on cesium concentration agrees with those of light absorbance, transient photovoltage, and PL decay time constants (see Figure 6 and Figure 8). Subsequently, it is obvious that performance of the perovskite solar cells is strongly determined by the quality of the perovskite films mainly represented by a long carrier lifetime and strong light absorption.

## 4. Conclusions

We experimentally investigated surface morphology, photoluminescence, and optoelectronic properties of perovskite Cs_x_(MA_0.17_FA_0.83_)_1−x_ Pb(I_0.83_Br_0.17_)_3_ films with different Cs dosage ranging from x = 0 to x = 0.6. Addition of small amount of Cs (x up to 0.1) leads to increased charge carrier lifetime and better performance of the perovskite solar cells (PCE = 20.2% in case of x = 0.1). At higher cesium concentration, x > 0.1, the carrier lifetime decreases and, as a result, PCE of the solar cells also drops down. An X-ray diffraction and SEM morphology studies of the perovskite films reveal that high dosage of Cs (x > 0.2) causes phase segregation which in turn leads to weaker light absorption due to formed CsPbI_3_ regions. The presented results provide a background for further improvement of material synthesis and device fabrication in the efforts of development of higher performance perovskite solar cells.

## Figures and Tables

**Table 1:** Elemental composition of the 30% CsI containing perovskite film surface, atomic % (oxygen and titanium values not included).

Point in Figure 3	Br	Sn	I	Cs	Pb
1	10.7	4.1	57.6	0.8	22.1
2	6.3	2.0	52.4	17.5	17.4

**Table 2:** Results of PL analysis.

		Cs Concentration, %				
		0	10	20	40	60
Band gap, eV (wavelength, nm)		1.61 (770)	1.62 (765)	1.64 (756)	1.63 (760)	1.62 (765)
*λ_max_*, nm		770	762	752	746	747
Gaussian	*λ_c_*_1_, nm	767	761	752	739	743
	*ω*_1_ (FWHM), nm	11 (26)	12 (28)	14 (33)	37 (87)	32 (75)
	*A*_1_, arb. u.	0.29	0.34	0.47	0.13	0.17
	*λ_c_*_2_, nm	775	770	756	746	747
	*ω*_2_ (FWHM), nm	23 (54)	25 (59)	23 (54)	18 (42)	17 (40)
	*A*_2_, arb. u.	0.73	0.69	0.53	0.88	0.83
Equation (1)	*A*, arb. u.	2.1	2.4	2.3	1.5	1.8
	*λ_c_*, nm	769	761	752	745	747
	*ω*, nm	19	13	15	31	25
	*ω*_1_, nm	10	11	10	10	12
	*ω*_2_, nm	15	17	13	11	11
Equations (4) and (5)	*A*, arb. u.	0.95	0.83	0.79	0.72	0.79
	*α*	9.5	59	1.3	1.7	1.2
	*β*	0.37	0.35	7E-8	0.24	0.19
	$\tau_{0}$, ns	7.6	0.54	2.7	4.7	6.4
	$\bar{\tau }$	698	2206	Inf.	108	117

**Table 3:** Photovoltaic parameters of the perovskite solar cells with different cesium concentration.

Cs, %	*V*_oc_, V	*J_sc_*, mA·cm^−2^	FF, %	PCE, %
0	1.13	20.2	64	14.6
10	1.11	23.6	77	20.2
20	1.09	20.8	74	16.8
40	1.01	18.3	71	13.1
60	0.96	16.0	70	10.8

## Data Availability

No new data were created or analyzed in this study. Data sharing is not applicable to this article.
